# Supplementary material for: IL1β/IL1R1/IRAK4 Drives Inflammatory Ovarian Cancer Seeding at the inflamed sites and Is Reversed by an IRAK4 inhibitor UR241-2
Source: bioRxiv. 2026 May 5:2026.04.30.722105. Preprint. [Version 1] doi: 10.64898/2026.04.30.722105 (PMC13174620; doi:10.64898/2026.04.30.722105)

bioRxiv preprint doi: https://doi.org/10.64898/2026.04.30.722105; this version posted May 5, 2026. The copyright holder for this preprint (which was not certified by peer review) is the author/funder, who has granted bioRxiv a license to display the preprint in perpetuity. It is made available under aCC-BY 4.0 International license.

# Supplementary Information

## IL1β/IL1R1/IRAK4 Drives Inflammatory Ovarian Cancer Seeding at the inflamed site and Is Reversed by UR241-2

John P. Miller[1], Kyu Kwang Kim[1], Cameron WA Snyder[1], Negar Khazan[1], Niloy A. Singh[1,2], Megan E. Boyer[1], Elizabeth Lamere[2], Myla Strawderman[3], Sonali Sharma[4], Ronald Lakony[2], Michelle Whittum[1], Mark Anderson[5], Rick Keenan[5], Elizabeth Pritchett[6], Cameron Baker[6], John Ashton[6], Manoj K. Khera[7], Michael R. Elliott[9], Christina M. Annunziata[10], Jeevisha Bajaj[4], Laura M. Calvi[2], Michael W. Becker[10], Rachael Rowswell-Turner[1*], Richard G. Moore[1]*, Rakesh K. Singh[1*]

[1]Department of Microbiology and Immunology, University of Rochester, Rochester, NY, USA,14642.
[2] Department of Medicine, University of Rochester Medical Center, Rochester, NY, USA, 14642.
[3] Department of Biostatistics and Computational Biology, University of Rochester Medical Center, Rochester, NY, USA,14642.
[4] Department of Biomedical Genetics, University of Rochester Medical Center, Rochester, NY, USA,14642.
[5]Empire Discovery Institute, University of Rochester, Rochester, NY, USA, 14642.
[6]Genomic Research Center, University of Rochester Medical Center, Rochester, NY, USA,14642,
[7]Presude Lifesciences Private Limited, Delhi, India.
[8]Department of Microbiology and Immunology, University of South Alabama, Mitchell Cancer Institute, Mobile, AL, USA, 36604.
[9] American Cancer Society, Atlanta, GA, USA, 30303.
[10]Previously at Department of Medicine, University of Rochester Medical Center, Rochester, NY, USA; and Indiana University Simon Comprehensive Cancer Center, Indianapolis, Indiana, USA, 46202.

**Corresponding authors**:

Rakesh K Singh PhD MBA (Primary contact): Rakesh_Singh@urmc.rochester.edu
Richard Moore MD: Richard_Moore@urmc.rochester.edu
Rachael Rowswell-Turner MD PhD: Rachael_Turner@urmc.rochester.edu

bioRxiv preprint doi: https://doi.org/10.64898/2026.04.30.722105; this version posted May 5, 2026. The copyright holder for this preprint (which was not certified by peer review) is the author/funder, who has granted bioRxiv a license to display the preprint in perpetuity. It is made available under aCC-BY 4.0 International license.

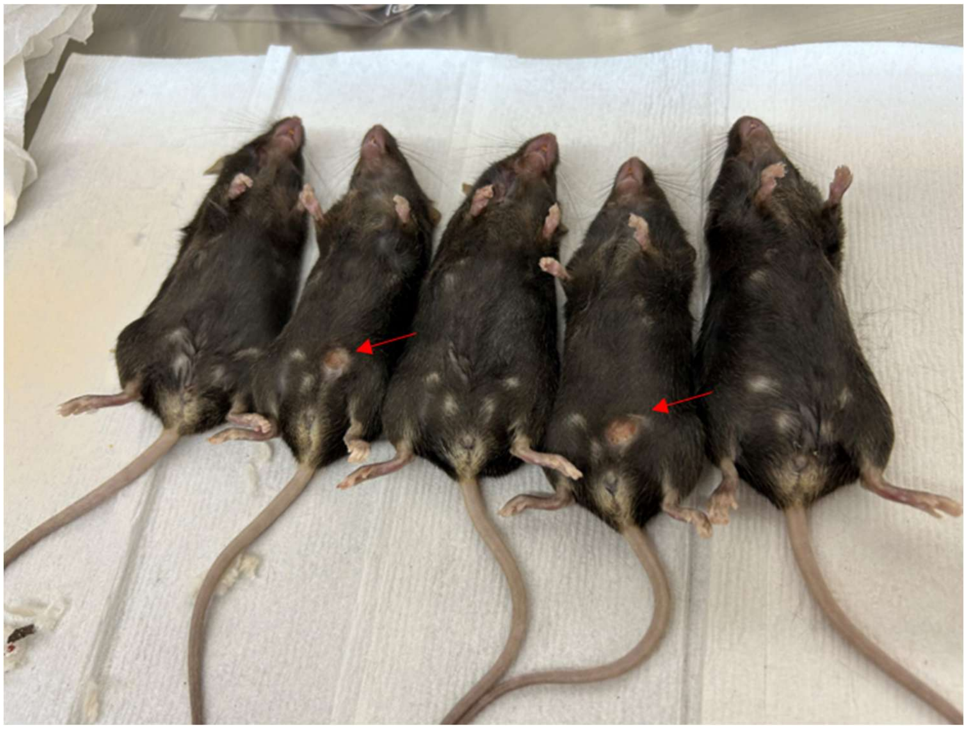

**Supplementary Figure-1:** HGS-3 murine high-grade serous EOC cells (3-4.5 million/per mice) were implanted intraperitoneally using 21-gauge needle in C57BL/6 WT and C57BL/6 Nlrp3$^{KO}$ mice. Mice were observed for 45-50 days and euthanized. Tumors formed on needle injury site, protruding at the skin as well as in the peritoneum and on the omentum, shown by red arrows, were isolated, weighed and frozen in liquid -nitrogen. Lavages via washing with sterile PBS(5mL) were also collected. The studies were repeated twice. A representative experiment is shown. Weights of the omental did not differ between C57BL/6 WT (Figure-1H) and Nlrp3$^{KO}$ mice. Similarly, the tumor sizes at the site of injury did not differ between C57BL/6 WT and Nlrp3$^{KO}$ mice.

bioRxiv preprint doi: https://doi.org/10.64898/2026.04.30.722105; this version posted May 5, 2026. The copyright holder for this preprint (which was not certified by peer review) is the author/funder, who has granted bioRxiv a license to display the preprint in perpetuity. It is made available under aCC-BY 4.0 International license.

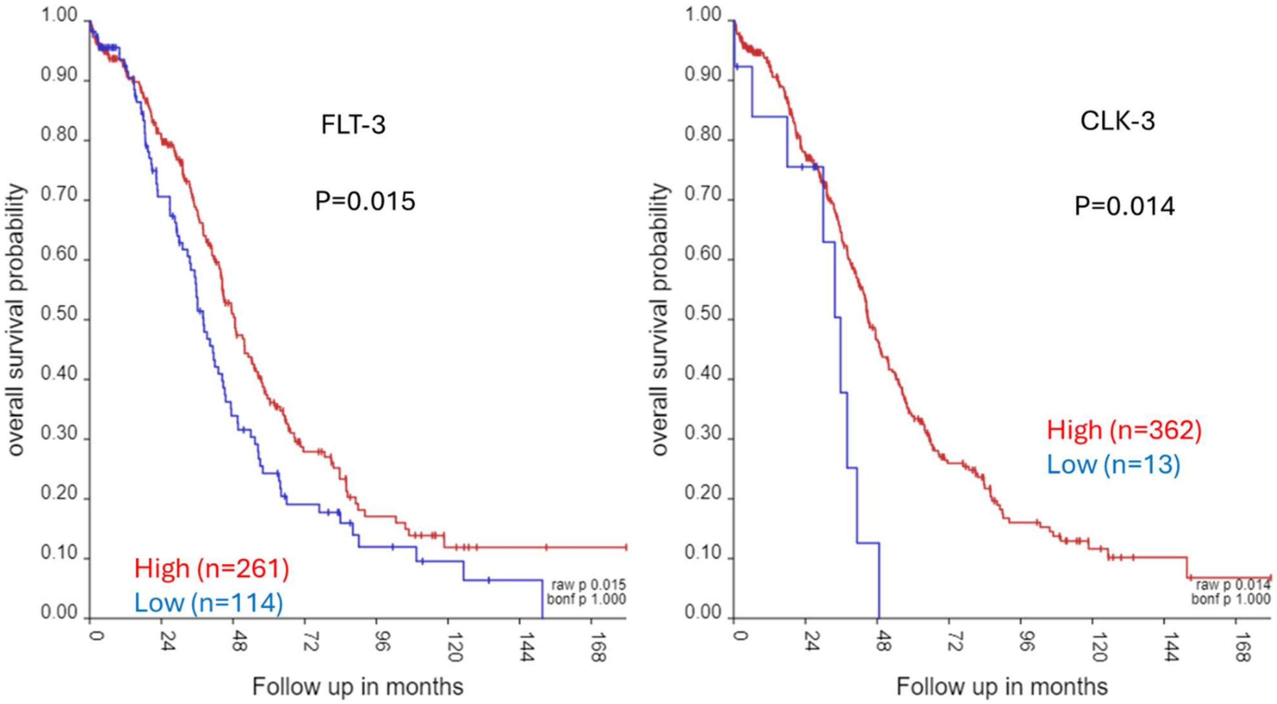


**Supplementary Figure-2:** Analysis of ovarian serous cystadenocarcinoma (2022-v32) microarray data (TCGA-381-tpm-gencode36) of ovarian cancer patients using R2 Genomics Analysis and Visualization Platform tools showed that FLT-3 and CLK-3 mRNA overexpression predicts poor survival.

bioRxiv preprint doi: https://doi.org/10.64898/2026.04.30.722105; this version posted May 5, 2026. The copyright holder for this preprint (which was not certified by peer review) is the author/funder, who has granted bioRxiv a license to display the preprint in perpetuity. It is made available under aCC-BY 4.0 International license.

A

| Compound ID | λ (nm) | Solubility (μM) |
|---|---|---|
| UR-241-2 | 280 | 29 |
| Verapamil | 280 | 89 |
| Tamoxifen | 280 | 4.3 |

B

| Project Compound ID | Human Liver Microsomes | | | Mouse Liver Microsomes | | |
|---|---|---|---|---|---|---|
| | $t_{1/2}$ (min) | $CL_{int}$ (μL/min/mg) | $E_H$ | $t_{1/2}$ (min) | $CL_{int}$ (μL/min/mg) | $E_H$ |
| EDI-238382 | 214 | 3.24 | 11% | 13.5 | 51.4 | 53% |
| Testosterone* | 21.8 | 31.8 | 56% | 7.59 | 365 | 89% |
| UR241-2 | 208 | 3.3 | 12% | 8.70 | 79.7 | 63% |
| Testosterone* | 11.3 | 61.5 | 71% | 6.83 | 406 | 90% |

C

| Compound ID | CYP $IC_{50}$ (μM) | | | | | | | |
|---|---|---|---|---|---|---|---|---|
| | CYP1A2 | CYP2B6 | CYP2C8 | CYP2C9 | CYP2C19 | CYP2D6 | CYP3A4M | CYP3A4T |
| UR-241-2 | >100 | >100 | 79 | 30 | 45 | >100 | 58 | 39 |
| Furafylline | 1.8 | | | | | | | |
| Ticlopidine Hydrochloride | | 0.093 | | | | | | |
| Montelukast Sodium Hydrate | | | 0.013 | | | | | |
| Sulfaphenazole | | | | 0.17 | | | | |
| (+)-N-3-Benzylnirvanol | | | | | 0.20 | | | |
| Quinidine | | | | | | 0.11 | | |
| Ketoconazole | | | | | | | 0.014 | 0.010 |
| Z' | 0.73 | 0.80 | 0.78 | 0.77 | 0.74 | 0.66 | 0.79 | 0.76 |
| $r^2$ | 1.0 | 0.99 | 0.99 | 0.99 | 1.0 | 0.99 | 1.0 | 1.0 |

| % control activity at 100 μM | | | | | | | |
|---|---|---|---|---|---|---|---|
| CYP1A2 | CYP2B6 | CYP2C8 | CYP2C9 | CYP2C19 | CYP2D6 | CYP3A4M | CYP3A4T |
| 68 | 67 | 42 | 28 | 33 | 62 | 49 | 40 |

D

| Project Compound ID | Protein Binding, % | | Recovery, % | |
|---|---|---|---|---|
| | Human Plasma | Mouse Plasma | Human Plasma | Mouse Plasma |
| *UR-241-2 | 97.4 ± 0.3 | 93.8 ± 0.8 | 92.1 | 55.1 |
| Propranolol | 73.7 ± 1.6 | 81.2 ± 1.3 | 100 | 115 |

E

| Compound ID | Lot | % Recovery | | $P_{app}$ ($x10^{-6}$ cm/s) | | Efflux Ratio | Permeability Classification | Significant Efflux |
|---|---|---|---|---|---|---|---|---|
| | | A-B | B-A | A-B | B-A | | | |
| UR-241-2 | 12-WEN-111-1 | 76.8 | 85.2 | 11.9 | 49.4 | 4.14 | High | Yes |

F

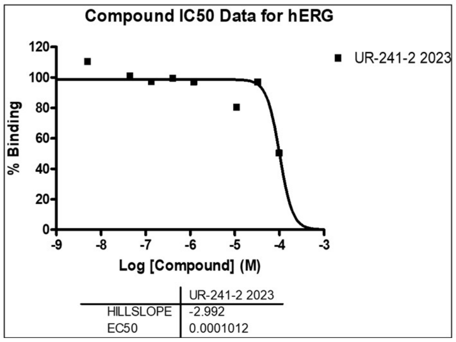


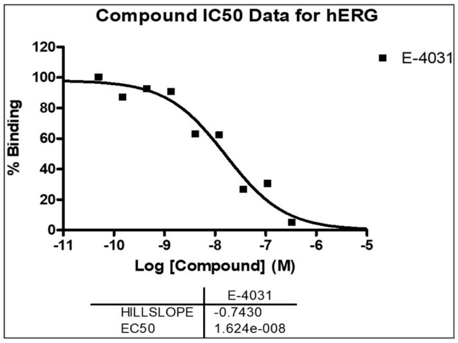


G

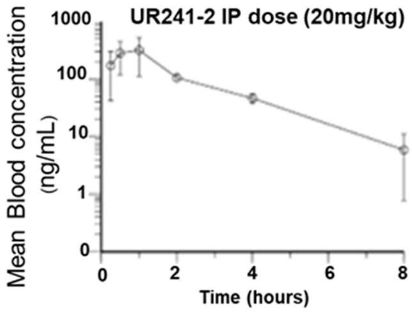

bioRxiv preprint doi: https://doi.org/10.64898/2026.04.30.722105; this version posted May 5, 2026. The copyright holder for this preprint (which was not certified by peer review) is the author/funder, who has granted bioRxiv a license to display the preprint in perpetuity. It is made available under aCC-BY 4.0 International license.

**Supplementary Figure-3**: (**A**): Kinetic solubility of UR241-1 is shown. (**B**): Quantified stability of UR241-2 in human and mouse liver microsomes is shown. (**C**): Inhibition of CYP450 isoforms by UR241-2 is quantified using HPLC. Controls used were furafyllin, Ticlopidine HCL, Montelukast sodium hydrate, sulfaphenazole, N-3-benznirvanol, Qunidine, ketoconazole. CYP isoforms affected by UR241-2 in terms of %-inhibition are shown. (**D**): Human and murine plasma protein binding of UR241-1 is shown in % units. Propranolol was used as a control. (**E**): CaCo-2 cell permeability of UR241-2 is shown. Efflux ratio of 4.14 indicates that UR241-2 faces significant efflux. (**F**): UR241-2 does not inhibit hERG. $IC_{50}$ is 101.2μM. E-4031 was used as control. $IC_{50}$ for E-4031 was $1.62e^{-08}$. (**G**): Pharmacokinetic (PK) of UR241-2 at 20mg/kg administered IP is shown. It is shown that ~8ng/ml concentration of UR241-2 is maintained up until 8$^{th}$ hour of monitoring.

bioRxiv preprint doi: https://doi.org/10.64898/2026.04.30.722105; this version posted May 5, 2026. The copyright holder for this preprint (which was not certified by peer review) is the author/funder, who has granted bioRxiv a license to display the preprint in perpetuity. It is made available under aCC-BY 4.0 International license.

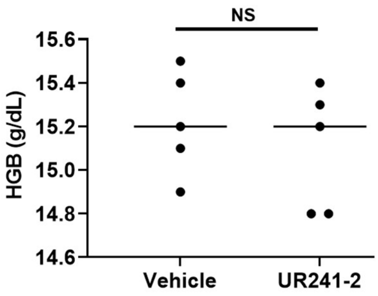


**Supplementary Figure-4**: Analysis of the peripheral blood showed that hemoglobin (HB) levels did not differ between the vehicle and UR241-2 treated mice.

bioRxiv preprint doi: https://doi.org/10.64898/2026.04.30.722105; this version posted May 5, 2026. The copyright holder for this preprint (which was not certified by peer review) is the author/funder, who has granted bioRxiv a license to display the preprint in perpetuity. It is made available under aCC-BY 4.0 International license.

| Reference /Cat | Manufacturer | Name | Other Name | Clone | Fluorochrome | Max Excitation | Max Emission | Laser |
|---|---|---|---|---|---|---|---|---|
| 565992 | BD Biosciences | CD3e | | 145-2C11 | BUV395 | 348 | 395 | UV |
| 364-0081-82 | eBioscience | CD8a | | 53-6.7 | BUV496 | | | UV |
| 612793 | BD Biosciences | CD69 | | H1.2F3 | BUV737 | 350 | 737 | UV |
| 749284 | BD OptiBuild | F4/80 | | T45-2342 | BUV563 | 351 | 561 | UV |
| 750042 | BD OptiBuild | CD192 | CCR2 | 475301 | BUV661 | 348 | 661 | UV |
| 568287 | BD Horizon | CD19 | | 1D3 | BUV805 | | | UV |
| 752299 | BD OptiBuild | CD279 | PD-1 | J43 | BUV615 | 350 | 616 | UV |
| 563053 | BD Horizon | CD45 | | 30-F11 | Brilliant Violet 605 | 407 | 605 | Violet |
| 564023 | BD Biosciences | CD25 | | PC61 | Brilliant Violet 785 | 408 | 786 | Violet |
| 101251 | Biolegend | CD11b | | M1/70 | Brilliant Violet 421 | 408 | 422 | Violet |
| 560458 | BD Horizon | CD11b | Gr1 | 1A8 | V450 | 404 | 448 | Violet |
| 560603 | BD Horizon | Ly6g | Gr1 | 1A8 | V450 | 404 | 448 | Violet |
| 560593 | BD Pharmingin | Ly-6C | | AL-21 | PE-Cy7 | 568 | 778 | Yellow-Green |
| 558091 | BD Biosciences | CD274 | PD-L1 | MIH5 | PE | 496 | 578 | Yellow-Green |
| 11-5773-82 | eBioscience | FoxP3 | | FJK-16s | FITC | 494 | 520 | Blue |
| 566504 | BD Horizon | CD11c | | HL3 | BB700 | | | Blue |
| | Biolegend | CX3CR1 | Fractalkine receptor | SA011F11 | PE-Dazzle594 | | | Blue |
| 552051 | BD Pharmingin | CD4 | | GK1.5 | APC-Cy7 | 650 | 775 | Red |
| 17-5321-82 | eBioscience | MHCII | I-A/I-E | M5/114.15.2 | APC | 650 | 660 | Red |
| 47-5932-82 | invitrogen | Ly6C | | HK1.4 | APC-eFluor 780 | | | |
| 25-2061-82 | invitrogen | CD206 | | MR6F3 | PE-Cy7 | 568 | 778 | Yellow-Green |
| L34959 | Invitrogen | LiveDead Yellow | | | | | | Violet |

**Supplementary data-5:** List and catalog details (reference no, manufacturer, name, other name, Clone, fluorochrome, max and min excitation, laser) of flow cytometry antibodies used in this study.

bioRxiv preprint doi: https://doi.org/10.64898/2026.04.30.722105; this version posted May 5, 2026. The copyright holder for this preprint (which was not certified by peer review) is the author/funder, who has granted bioRxiv a license to display the preprint in perpetuity. It is made available under aCC-BY 4.0 International license.

## Supplementary Data-6

### NMR and Mass spectrometry data for UR241-2:

$^{1}H$ NMR (400 MHz, $CDCl_3$), δ: 10.52 (s, 1H), 8.51 (d, *J* = 8.7 Hz, 1H), 8.21 (d, *J* = 6.6 Hz, 1H), 8.04 – 7.93 (m, 2H), 7.72 (d, *J* = 2.1 Hz, 1H), 6.98 (d, *J* = 2.1 Hz, 1H), 6.60 (dd, *J* = 8.8, 2.5 Hz, 1H), 6.56 (d, *J* = 2.4 Hz, 1H), 4.01 (s, 3H), 3.86 – 3.77 (m, 4H), 3.21 – 3.11 (m, 4H). LCMS (m/z): 428.3 $[M+H]^+$.

### NMR and Mass spectrometry data for PSP-0099:

$^{1}H$ NMR (400 MHz, $CDCl_3$), δ: 10.51 (s, 1H), 8.48 (d, J = 9.3 Hz, 1H), 8.21 (d, J = 7.2 Hz, 1H), 8.02 – 7.92 (m, 2H), 7.72 (d, J = 1.9 Hz, 1H), 6.98 (s, 1H), 6.60 (d, J = 6.5 Hz, 2H), 4.00 (s, 3H), 3.57 – 3.50 (m, 3H), 2.86 – 2.77 (m, 4H). LCMS (m/z): 396.3 $[M+H]^+$.

### NMR and Mass spectrometry data for PSP-0100:

$^{1}H$ NMR (400 MHz, $CDCl_3$), δ: 10.51 (s, 1H), 8.49 (d, J = 8.7 Hz, 1H), 8.21 (d, J = 7.3 Hz, 1H), 7.97 (dt, J = 15.3, 7.5 Hz, 2H), 7.72 (d, J = 1.5 Hz, 1H), 6.98 (s, 1H), 6.64 (dd, J = 8.8, 2.4 Hz, 1H), 6.60 (d, J = 2.4 Hz, 1H), 3.99 (d, J = 10.2 Hz, 3H), 3.95 (dd, J = 10.2, 3.2 Hz, 2H), 3.54 (dd, J = 18.1, 3.7 Hz, 2H), 3.00 – 2.85 (m, 4H). LCMS (m/z): 412.2 $[M+H]^+$.

## Supplementary data-7

### Gating strategy

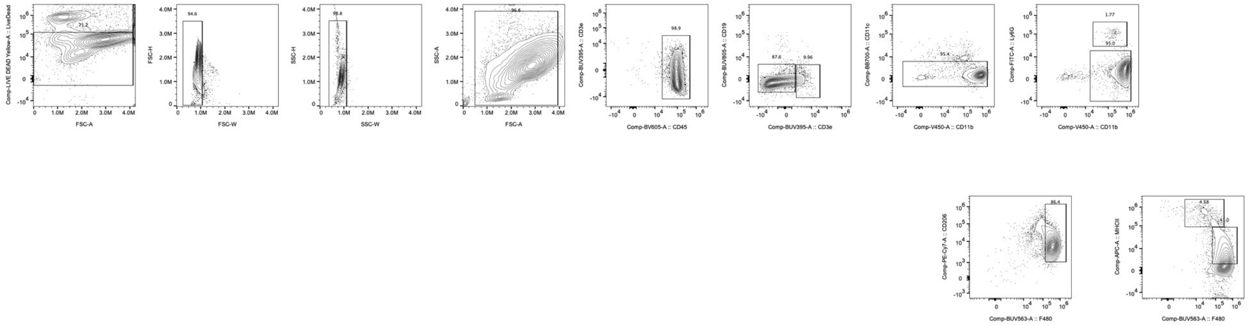

Supplement: 1 [file NIHPP2026.04.30.722105v1-supplement-1.pdf]
